# Tea tree recognition based on multi-source satellite data across Southwest China

**DOI:** 10.3389/fpls.2026.1801301

**Published:** 2026-05-14

**Authors:** Nan Cong, Rongrong Zhao, Yuxin Qiu, Chuang Zhao

**Affiliations:** 1 Lhasa Plateau Ecosystem Research Station, Institute of Geographic Sciences and Natural Resources Research, Chinese Academy of Sciences, Beijing, China; 2 College of Resources and Environmental Sciences, China Agricultural University, Beijing, China

**Keywords:** classification accuracy, crop distribution, machine learning, remote sensing, tea trees

## Abstract

The tea plant (Camellia Sinensis), as the world’s most popular non-alcoholic beverage, underscores the importance of precise and timely spatial data for industry insights and sustainability. Yet, the accurate delineation of spectrally similar vegetation types, notably tea trees, continues to elude conventional methods. This study innovatively integrates Sentinel-1 radar data with Sentinel-2 imagery to effectively overcome optical observation limitations imposed by Yunnan’s cloudy climate, particularly during non-growing seasons (e.g., February). By systematically analyzing annual phenological dynamics, we quantitatively identified April as the optimal temporal window for discriminating tea trees from spectrally similar vegetation, such as rubber and natural forests. Furthermore, optimizing the model through a feature selection process to eliminate redundant features significantly enhanced the overall classification accuracy from 87.1% to 89.1%. This study could assistant monitoring the crop dynamics and timely respond to cultivation activity.

## 1 Introduction

The tea plant (*Camellia sinensis*), originating in southwestern China, is the world’s most popular non-alcoholic beverage (Yang et al., 2023) and holds significant botanical and economic importance (Xia et al., 2020). As the leading global producer, China accounted for 46.8% of global tea production (2.93 million tons) in 2020, a figure driven by the crop’s high profitability and expanding global demand (Liu et al., 2018; Yao and Chen, 2012; National Bureau of Statistics, 2021; Su et al., 2017). Consequently, securing timely and precise data on tea plantation areas and spatial distribution is crucial for industry analysis, regulatory measures, optimizing regional production, and fostering sustainable development. However, achieving accurate large-scale mapping faces distinct challenges, particularly in regions like Yunnan Province. Yunnan tea trees are diverse and often exhibit a tall arbor form, contrasting with the shrub-dominated patterns found elsewhere (Xu and Zhang, 2009). In the province’s complex ecological environment, these arbor tea trees are easily confused with natural woodlands, posing a significant obstacle to traditional identification methods.

Remote sensing technology offers a feasible means to precisely and rapidly obtain information on crop planting areas (Löw et al., 2013). So far, numerous studies have been performed on landcover classification using satellite observations, for example, the spatial resolution of satellite images used in these studies ranges from hundreds of meters to less than 1 m (Ren et al., 2019). Most classifications have focused on major food crops, with accuracy improving over time (Li et al., 2019). Whether this technology and its methods can be directly applied to non-major crops largely depends on the specific characteristics of these crops and the monitoring requirements (Löw and Duveiller, 2014). In general crop remote sensing classification, mainstream methodologies primarily leverage the distinct electromagnetic reflection properties of vegetation. These approaches typically integrate multi-spectral analysis (visible, NIR, and SWIR bands), a variety of vegetation indices (e.g., NDVI, EVI, SAVI) to enhance biophysical parameter discrimination, and phenological dynamics to identify optimal observation windows where spectral differences between crop types are maximized (Li et al., 2018). Increasingly, data fusion strategies combining optical imagery with Synthetic Aperture Radar (SAR) are employed to mitigate weather-related limitations and incorporate structural information (Kang et al., 2023).

Despite these advancements, tea plantation identification presents unique necessities and challenges that distinguish it from other crop types. Unlike staple grains which often have distinct seasonal bare-soil phases, tea plants are evergreen woody perennials cultivated in tropical and subtropical regions. Their spectral features frequently overlap with those of shrubs, natural forests, and other woody vegetation, making discrimination difficult using single-phase optical imagery alone (Kang et al., 2023). This challenge is exacerbated in Yunnan, where the tall arbor form of tea trees increases spectral confusion with natural forest canopies (Dihkan et al., 2013). Furthermore, existing research on tea identification is often limited to local scales using hyperspectral data; such approaches face hurdles in large-scale application due to complex data processing, high computational demands, and lower algorithmic efficiency (Melián et al., 2021; Bioucas-Dias et al., 2013). While some studies utilize phenological information, they often rely on prior knowledge (e.g., pruning periods) without systematically identifying the optimal observation phases for extensive plantation areas (Kang et al., 2023).

Compared to the satellite data with low spatial resolution, medium and high-resolution satellites such as Sentinel-2 have the ability to capture changes on the Earth’s surface more precisely and support land monitoring services. These images are commonly used in agricultural observation research, especially when data fusion is not required. Meanwhile, Synthetic Aperture Radar (SAR), with its unique capability to operate independently of light and weather conditions (Franceschetti and Lanari, 1999), has lately garnered attention for its potential to mitigate the vulnerability of optical remote sensing to cloud cover and shadow obstruction.

Objects’ electromagnetic reflection is the base for remote sensing imaging, which is received by sensors carrying on satellite and then transferred to visual images. Ground objects exhibit unique reflection characteristics due to their inherent properties, resulting in distinctive spectral characteristics of reflected and absorbed sunlight, allowing the use of spectral characteristics to identify land cover types (Xie et al., 2008). Crop spectral characteristics at different wavelengths vary depending on crop types and their growth stages in agricultural fields (Gates et al., 1965; Swaminathan et al., 2022). The radiation spectrum of the same land cover in remote sensing images captured at different time phases changes due to variations in sunlight, temperature, humidity, and other natural factors, leading to deviations in classification results (Lambin and Strahlers, 1994). Thus, in addition to enhancing the spatial and time-phase resolution, the selection of the best phase in remote sensing classification is a critical step in crop monitoring and yield estimation (Wei et al., 2023). Previous research has extensively investigated the application value of phenological information in economic crops such as cotton, fruit trees, and staple grains, notably achieving significant advancements in identifying crop growth cycles and enhancing classification accuracy (Qian, 1998; Xing et al., 2020; Qi et al., 2008). In contrast, while studies on tea plants have touched upon the utilization of phenological information, they largely rely on prior knowledge, simply incorporating the period of severe pruning as a key phenological characteristic into classification processes (Zhu et al., 2019; Kang et al., 2023). To date, no systematic study has explored the best observation phase tailored to extensive tea plantation areas. Hence, performing a detailed analysis of phenological sensitivity for tea plants can pinpoint the most suitable observation periods within their growth cycle, thereby maximizing the identification effectiveness and classification precision of remote sensing data.

This study focused on Yunnan Province, and Yunnan tea trees are diverse, characterized by wild arbor tea trees (Xu and Zhang, 2009). They exhibit a tall arbor form, which contrasts with the shrub-dominated tea plantation pattern in many regions. This characteristic poses a challenge to the identification of Yunnan tea trees, especially in the complex and diverse ecological environment, which is easily confused with woodlands. Therefore, we used spectral identification in combination with radar bands to study the characteristic reflectance of the plant itself, combined with the characteristics of the phenological period, to distinguish tea trees from other woodland-type vegetation more effectively. Optical data from Sentinel-1 C-band and Sentinel-2 were fused to analyze the spectral and vegetation index characteristics at different growth stages of tea plants and other land use types. The Random Forest algorithm was employed to extract tea plantation distribution for each month in 2023. we further investigated the impact of remote sensing images on the accuracy of tea plantation extraction and changes in tea plantation areas. Finally, best time-phase phases and features were selected to improve classification accuracy. Our methodology was primarily executed in the Google Earth Engine (GEE) environment, providing convenient access to simplify data and process large volumes of satellite data efficiently. Unlike approaches centered on individual tree detection, this research prioritizes regional-scale tea plantation monitoring, offering valuable spatial data products to support policy formulation, industrial oversight, and environmental modeling, thereby reinforcing its scientific contribution to sustainable agricultural development.

## 2 Materials and methods

### 2.1 Study area

**Figure 1:**
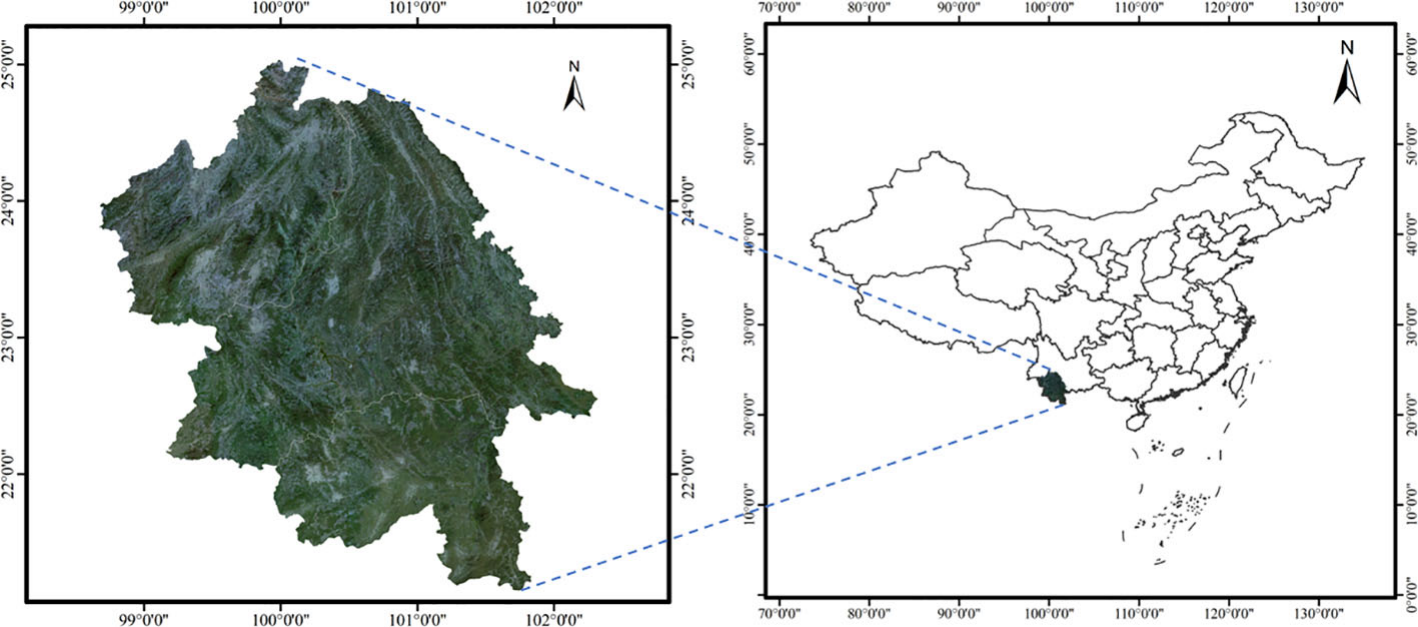
The study area.

Yunnan Province, situated between latitudes 20°8′ and 29°16′N, and longitudes 97°31′ to 106°12′E. The elevation rises from 76 meters in the southeast to a peak of 6,740 meters in the northwest (Yang et al., 2023). This study focuses on the southwestern part of Yunnan, encompassing Lincang City, Pu’er City, and Xishuangbanna Dai Autonomous Prefecture (**Figure 1**). This area includes varied terrain with significant elevation gradients, encompassing low mountains, hills, valleys, and mountainous landscapes, nurturing a rich array of ecosystems and biodiversity. The southwestern Yunnan has about six thousand years of tea cultivation, with renowned teas such as Pu’er and Dianhong originating here. The tea trees exhibit a distinct dormant phase during their growth cycle, and the harvest starts in early March (Yunnan Rural Executive College, 2012). the picking period lasting around 6 to 8 months (Sun et al., 2021).

### 2.2 Data and preprocessing

The images and preprocessing required for this study were all based on the GEE, including optical data from the Sentinel-2 A/B multispectral instrument (MSI) and the Sentinel-1 C-band. Band information for the two types of remote sensing data sources is presented in **Table 1**. To ensure the consistency and reliability of the multi-source remote sensing data, a series of preprocessing procedures were applied. For Sentinel-2 imagery, cloud and cirrus contamination were masked using the QA60 band by filtering out pixels with cloud-related bit flags (bits 10 and 11). The raw digital numbers from the Level-1C product were subsequently scaled by a factor of 0.0001 to convert them to top-of-atmosphere (TOA) reflectance. Regarding Sentinel-1 imagery, the COPERNICUS/S1_GRD product was utilized, which is radiometrically calibrated by default in the Google Earth Engine (GEE) platform.

**Table 1:** Band information of images employed in the research.

Sensors	Band		Wave length (μm)	Resolution (m)
Sentinel-2 MSI	Band 2	Blue	0.458-0.523	10
	Band 3	Green	0.543-0.578	10
	Band 4	Red	0.650-0.665	10
	Band 8	Near-infrared	0.785-0.900	10
	Band 11	Short-wave infrared 1	1.565-1.655	20
	Band 12	Short-wave infrared 2	2.100-2.280	20
Sentinel-1	VV	Dual-band cross-polarization, vertical		10
	VH	transmission/horizontal receiver		10

Sentinel-2 carries a Multispectral Imager (MSI) that covers 13 spectral bands with a swath width of 290 kilometers. Considering both Sentinel-2A and 2B, it can provide images at spatial resolutions of 10, 20, and 60 meters, with a revisit period of 5 days. In this study, Sentinel series data with a spatial resolution of 10 meters was used, utilizing the Blue, Green, Red, Near-Infrared (NIR), and two Short-Wave Infrared (SWIR1, SWIR2) spectral bands from Sentinel-2. To maximize the use of imagery, pixels void due to cloud obscuration were filled using the average value of pixels from monthly composite image data. Processed pixels were then used to compute the surface parameters required for following classification. Given the reduced cloud influence in monthly composites, we opted for imagery of southwestern of Yunnan Province from January to December 2022, for tea tree classification studies.

The Sentinel-1 SAR Ground Range Detected (S1_GRD) product was used in this study. It constitutes a Level-1 image dataset following Doppler centroid estimation, single-look complex (SLC) focusing, and post-processing. Each image contains four bands corresponding to four polarization combinations: horizontal transmit/horizontal receive (HH), horizontal transmit/vertical receive (HV), vertical transmit/vertical receive (VV), and vertical transmit/horizontal receive (VH), all at a resolution of 10 meters (Tan et al., 2019). This study primarily employed the VH and VV bands from Sentinel-1, further preprocessing each scene image through the Sentinel-1 toolbox available within the GEE cloud computing platform for tasks such as orbit parameter calibration, thermal noise removal, radiometric calibration, and terrain correction (Fei et al., 2022). Given the susceptibility of SAR data quality to imaging geometry, with pixels farther from the image center generally containing more pronounced noise, a solution was implemented in this research. Preprocessed SAR data was processed monthly, with each pixel having its signal values over the entire month aggregated into a median statistic. This approach aimed to filter out noise artifacts caused by excessive sensitivity of the observations to both imaging angles and surface conditions (Tan et al., 2019). Ultimately, a spatially resolved 10-meter and 250-kilometer-wide monthly composite product was obtained.

### 2.3 Sample acquisition for six dominant land cover types

Yunnan tea tree varieties can be broadly classified into three categories: wild arbor tea trees, cultivated ancient wild tea trees, and plantation tea (Xu and Zhang, 2009). Among these species, cultivated ancient wild tea trees and plantation tea are typically managed under a shrub tea garden regime. These tea trees, with their distinct textural characteristics, were predominantly selected as sampling targets during our process.

Taking into account the distribution of land types in the study area, the land cover is classified into five major categories, including tea plantation, rubber plantation, natural forest (including the forest-grass ecotone), water, farmland, and building land. In the intricate mountainous topography, the distinction between tea plantations, rubber forests, and natural forests often presents a challenge. To tackle this issue, we capitalized on the high-resolution remote sensing imagery resources furnished by Google Earth, integrating the employment of the Baidu Maps API interface technology. A series of targeted keywords, such as “tea plantation”, “tea farmer”, “tea garden”, “tea factory”, “tea estate”, and “tea planting”, were used in combination with Baidu Maps API searches, which initially yielded 243 tea gardens (refer to **Supplementary Table S1** for details). Through subsequent visual interpretation and manual verification, the dataset was expanded and refined, ultimately extracting precise geographical coordinates for 471 tea gardens. To address sampling bias arising from the overrepresentation of low-elevation areas, this study explicitly incorporates tea plantations from mountainous regions of Yunnan to ensure a more comprehensive analysis. To improve data precision and relevance, we undertook the conversion of these tea garden coordinates, originally expressed in the BD09 coordinate system and obtained via the Baidu Maps API, into the globally accepted WGS84 coordinate system. Each coordinate point was followingly subjected to scrutiny within the Google Earth platform, with sample features meticulously scrutinized and either discarded or augmented via visual interpretation. Ultimately, by leveraging ArcGIS 10.2, we uniformly transformed the processed data format and uploaded it onto the GEE cloud computing platform for detailed data processing. This culminated in the selection of 542 tea tree samples, 113 forest samples, 121 rubber tree samples, 78 waterbody samples, 113 farmland samples, and 56 construction land samples.

**Figure 2:**
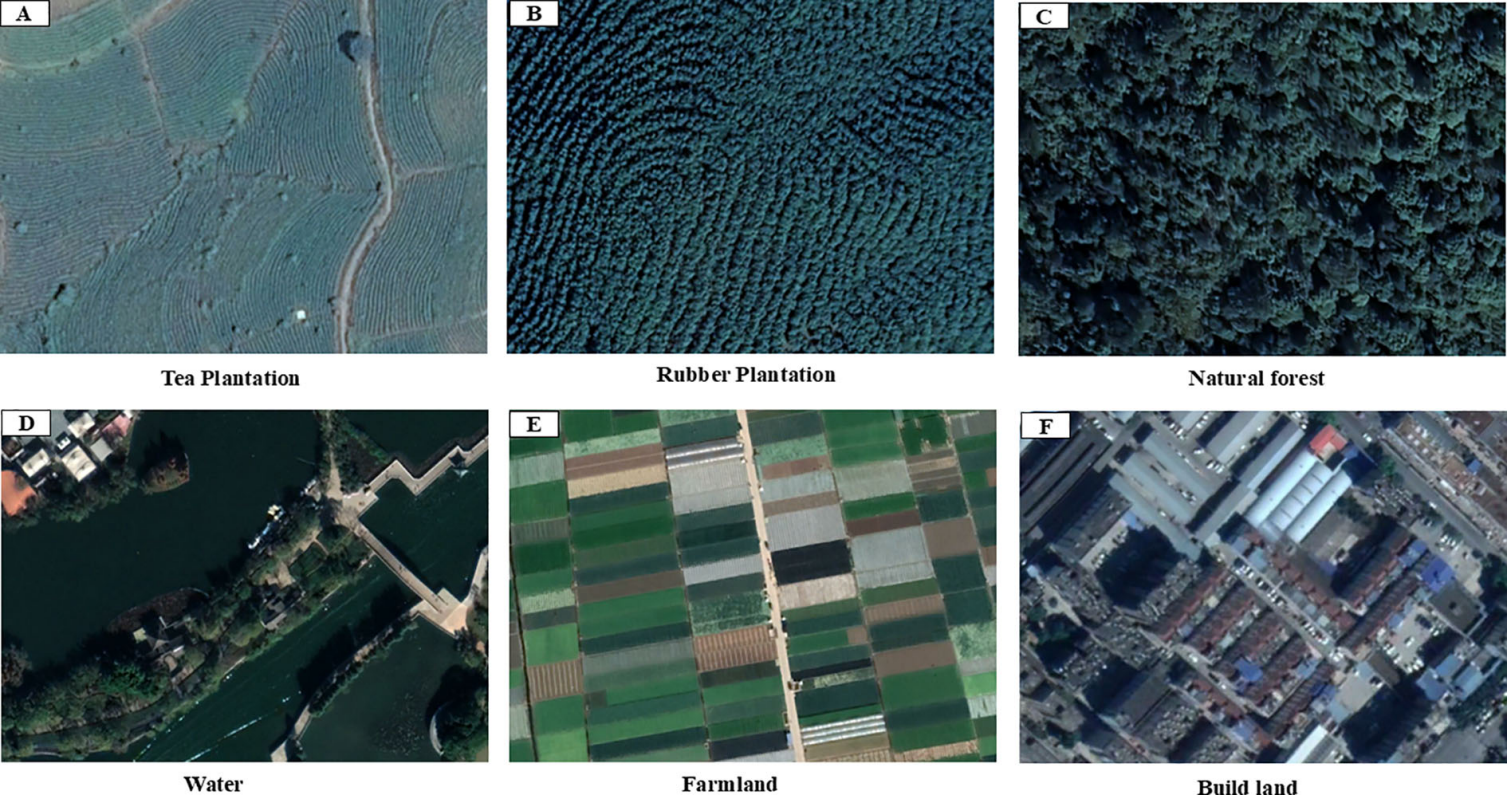
Sample feature illustration on imagery from Google Earth.

**Figure 3:**
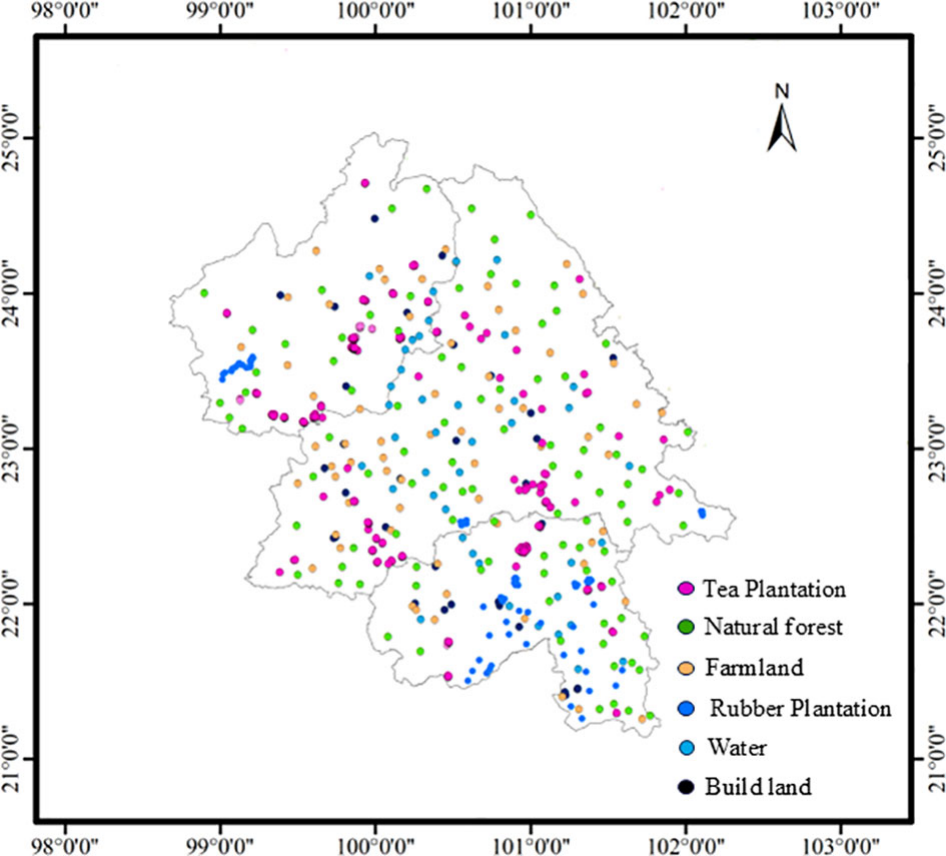
Distribution of samples in southwestern Yunnan Province, China.

These samples manifest distinct characteristics within the high-resolution remote sensing imagery supplied by Google Earth (**Figure 2**), allowing for precise discrimination based on *a priori* knowledge and visual interpretation. All chosen samples were systematically partitioned into training and validation sets in a scientifically sound manner. When implementing the random forest model, we adhered to the principle of stratified random sampling, dedicating 70.0% of the aggregate sample population to serve as training samples, while the residual 30.0% constituted the validation samples. The spatial distribution of sample points within the study area is illustrated in **Figure 3**.

### 2.4 Feature selection for tea tree pixels

**Figure 4:**
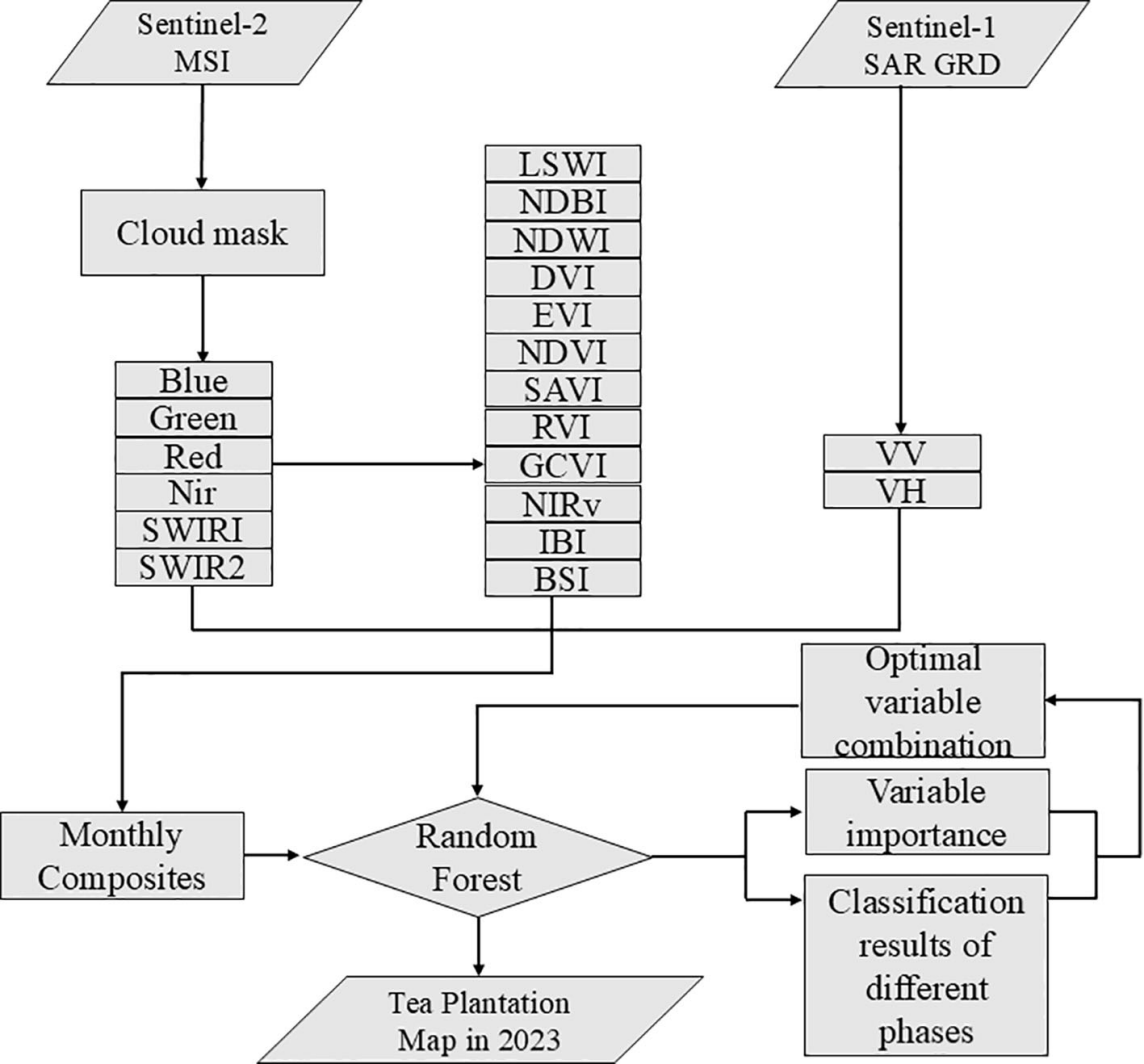
Flowchart of tea plantation extraction using random forest. “Blue”, “Green”, “Red”, “Nir”, “SWIR1”, “SWIR2”, “VH”, and “VV” are feature bands in Sentinel-2 MSI and Sentinel-1. “LSWI”, “NDBI”, “NDWI”, “DVI”, “EVI”, “NDVI”, “SAVI”, “RVI”, “GCVI”, “NIRv”, “IBI”, and “BSI” are abbreviations for the spectral index features used in this study.

Tea plantations were extracted using spectrum, vegetation index and SAR. This study employs feature-level fusion, which enables the integration of multi-source information at the feature extraction stage, thereby enhancing the discrimination of tea plantations across heterogeneous landscapes. This study harnesses the strengths of the GEE cloud computing platform in processing and integrating diverse, high-dimensional data attributes to calculate the vegetation indices for the six aforementioned object categories at different phases. We used 12 spectral indices to distinguish tea plantations from other land-use types in the study area. Adding these features to the tea plantation extraction process can thus make up for the lack of spatial information for spectral features and improve classification accuracy. Specific details can be found in **Figure 4**; **Table 2**.

**Table 2:** Information on spectral indices.

Spectral indices	Description	Formula
LSWI	Land surface water index	LSWI=(NIR−SWIR1)/(NIR+SWIR1)
NDBI	Normalized difference building index	NDVI=(SWIR2−NIR)/(SWIR2+NIR)
NDWI	Normalized difference water index	NDWI=(NIR−SWIR1)/(NIR+SWIR1)
DVI	Difference vegetation index	DVI=NIR−RED
EVI	Enhanced vegetation index	$\text{EVI}=2.5*(\text{NIR}-\text{RED})/(\text{NIR}+{\text{C}}_{1}\text{RED}-{\text{C}}_{2}\text{BLUE})$ ${\text{C}}_{1}=6.0;{\text{C}}_{2}=7.5;l=1.0$
NDVI	Normalized difference vegetation index	NDVI=(NIR−RED)/(NIR+RED)
SAVI	Soil adjusted vegetation index	SAVI=(NIR−RED)(1+L)/(NIR−RED+L) L=0.5
RVI	Ratio vegetation index	RVI=RED/NIR
GCVI	Green chlorophyll vegetation index	GCVI=NIR/GREEN−1
NIRv	Near-Infrared Reflectance of Vegetation	${\text{NIR}}_{\text{v}}=\text{NDVI}*\rho_{\text{NIR}}$
IBI	Index-based built-up index	IBI=[NDBI−(SAVI+MNDWI)/2]/[NDBI+(SAVI+MNDWI)/2]
BSI	Bare soil index	$\text{BSI}=[\left(\text{MIR}+\text{RED}\right)-(\text{NIR}+\text{BLUE})]/[\left(\text{MIR}+\text{RED}\right)+(\text{NIR}+\text{BLUE})\}$

NIR, Near Infrared; SWIR1, Shortwave Infrared 1; SWIR2, Shortwave Infrared 2; RED, Red Band; BLUE, Blue Band; GREEN, Green Band; MIR, Middle Infrared.

### 2.5 RF algorithm

The random forest (RF) algorithm is an efficient spectral classification method, known for its fast computation speed and ability to handle data at different scales, which have been widely used in landcover classification (Akar and Güngör, 2012; Jin et al., 2018). Considering Yunnan’s complex and varied terrain, and intermixed distribution with other vegetation types (such as forests, shrubs, and crops), the Random Forest algorithm was chosen for its ability to handle high-dimensional data with potentially complex nonlinear relationships among features, enhancing the model’s generalization capacity in complex environments (Hao et al., 2015). Within the GEE environment, we employed the RF classifier implemented using the SMILE library (ee.Classifier.smileRandomForest), configuring its key parameters.

One critical parameter of the Random Forest algorithm is the number of decision trees. Generally, increasing the number of decision trees improves overall model performance (Fawagreh et al., 2014). However, increasing the number of decision trees also raises computational costs. Setting the number of trees in a Random Forest to 100 is commonly regarded as a reliable choice in literature (Zhu et al., 2019), offering good classification performance in most cases while keeping computational resource consumption in check. The Random Forest model was configured with 100 decision trees (n_estimators), as preliminary tests showed accuracy plateaued beyond this point. To optimize performance and prevent overfitting, we set the maximum tree depth (max_depth) to None and the number of features per split (max_features) to $\sqrt{20}$. Additionally, the bagging fraction was set to 0.8, meaning 80% of samples were randomly drawn with replacement for each tree. These parameters ensured a balance between computational efficiency and classification accuracy.

Random Forests can automatically calculate the contribution of each feature to the model’s predictive capability, known as feature importance (Zhou et al., 2021). In this study, we selected a set of 20 feature variables consisting of 6 spectral features, 12 vegetation index features, and 2 synthetic aperture radar (SAR) features (see **Table 3**). To identify the best feature subset, we employed the feature importance ranking method of Random Forests to screen the feature set.

**Table 3:** Feature variable settings for the random forest model.

Feature properties	Feature description
Spectral feature	Blue, Green, Red, Near-infrared, Short-wave infrared 1, Short-wave infrared 2
vegetation index feature	LSWI, NDBI, NDWI, DVI, EVI, NDVI, SAVI, RVI, GCVI, NIRv, IBI, BSI
SAR feature	VH, VV

**Figure 5:**
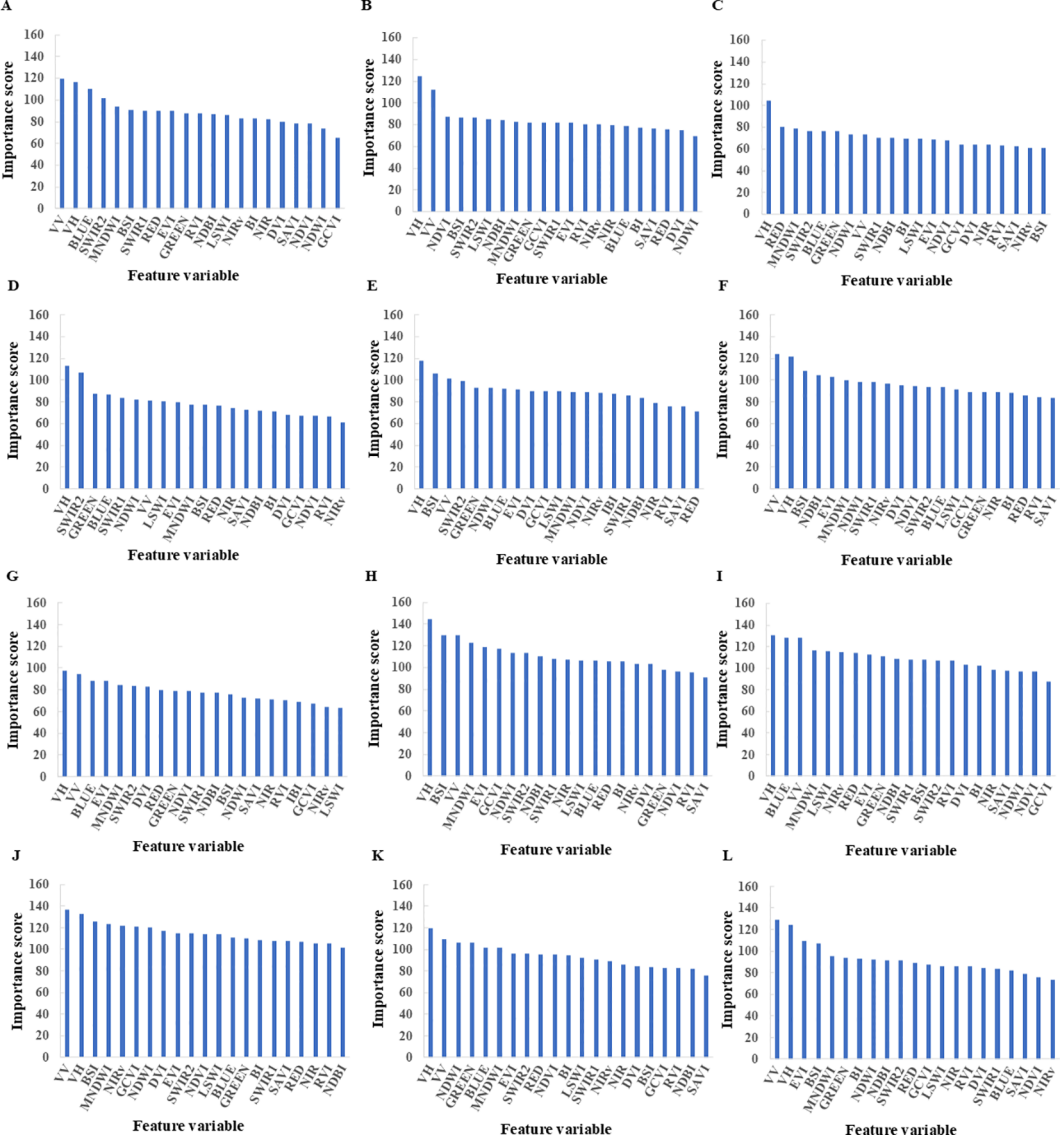
Sorting of the initial features based on importance cross different phases. **(A)** January 2023; **(B)** February 2023; **(C)** March 2023; **(D)** April 2023; **(E)** May 2023; **(F)** June 2023; **(G)** July 2023; **(H)** August 2023; **(I)** September 2023; **(J)** October 2023; **(K)** November 2023; **(L)** December 2023.

Although Random Forests are generally robust to multicollinearity in terms of prediction accuracy, high correlations among variables can dilute feature importance scores, causing correlated predictors to share importance and appear less significant individually. To address this potential issue, we employed an importance-based feature selection strategy. As illustrated in **Figure 5**, we ranked all variables according to their importance metrics. By retaining only the top-performing features and excluding those with marginal contributions, we effectively eliminated redundant variables caused by multicollinearity. This approach ensured that the final optimal subset contained the most representative and non-redundant predictors for tea plantation mapping.

## 3 Results and discussion

### 3.1 Spectral differentiation between tea trees and other land covers

**Figure 6:**
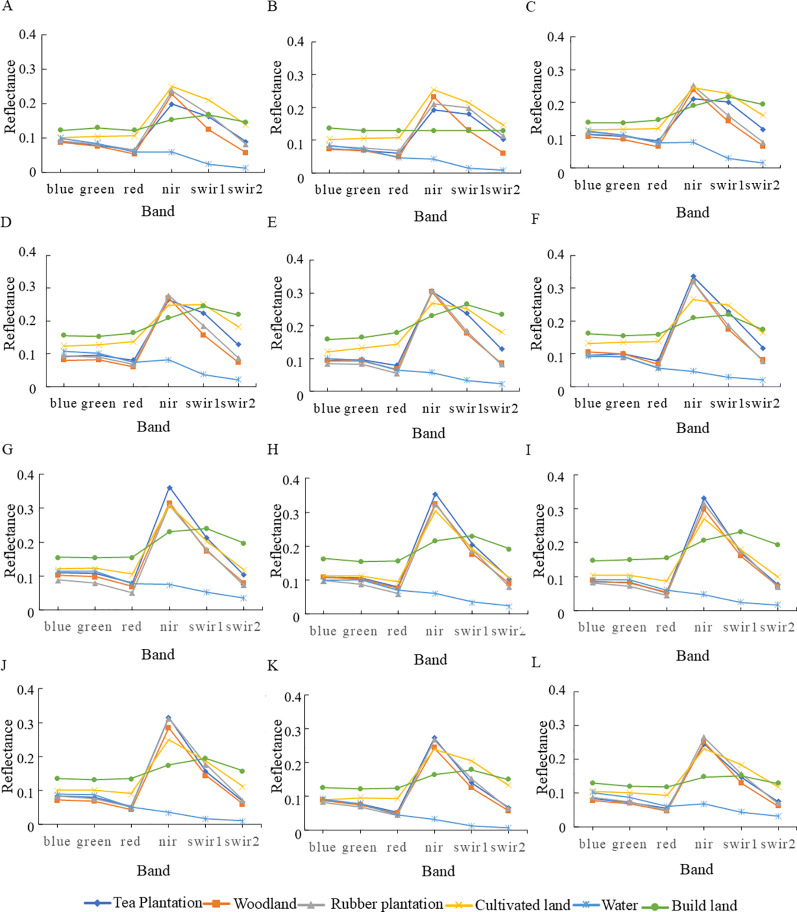
Spectral characteristic changes of tea in different phases. **(A)** January 2023; **(B)** February 2023; **(C)** March 2023; **(D)** April 2023; **(E)** May 2023; **(F)** June 2023; **(G)** July 2023; **(H)** August 2023; **(I)** September 2023; **(J)** October 2023; **(K)** November 2023; **(L)** December 2023.

Plant leaves exhibit strong absorption bands in the visible light range (400-700μm), including two absorption valleys near blue light at around 0.45μm and red light at around 0.65μm, as well as a reflection peak near green light at around 0.55μm (Mahlein et al., 2013). In contrast, the near-infrared range (0.7-1.3μm) represents a wavelength band where plant leaves exhibit strong reflection. From a single temporal perspective, **Figure 6A** illustrates that in January 2023, tea trees exhibited a significant difference in near-infrared spectral reflectance compared to other land cover types. Whereas in the shortwave infrared (SWIR1 and SWIR2) bands, tea trees display a certain degree of distinction from all land cover types except rubber trees. tea trees exhibit distinct reflectance characteristics in the SWIR range compared to all other land cover types (**Figures 6B–E**), with tea tree reflectance in the near-infrared band being lower than that of forests, rubber trees, and croplands in February and March. From June to September (**Figures 6F, I**), tea tree near-infrared reflectance is clearly higher than that of all other land cover types. From October to December, tea tree SWIR reflectance is higher than that of forests but lower than that of rubber trees and croplands, with the differences being less pronounced than in previous months. In summary, the spectral reflectance of tea trees in February and March shows the most pronounced differences compared to other land cover types.

From the seasonal dynamics in the spectral curves of tea plantations, **Figures 6A–C** show that from January to March 2023, tea trees exhibited a pattern of initially declining then ascending reflectance across the visible spectrum and near-infrared bands, while displaying a marked increase in the shortwave infrared. Comparing **Figures 6–F**, tea trees exhibit a significant drop in visible (Band 1~Band 4) reflectance in April, stabilizing thereafter and demonstrating a slight upward trend from June to July. Their near-infrared reflectance shows a marked and sustained increase, peaking in July; in the shortwave near-infrared region, their reflectance experiences a sharp increase followed by a gradual decline, with May being the maximum for this stage. **Figures 5H, L** reveal that from August to December 2023, tea tree visible band reflectance dips in September before settling into a relatively steady pattern, while near-infrared reflectance shows a clear decline. Tea tree shortwave near-infrared reflectance gradually decreases from August to November, with an increase in December.

### 3.2 Differentiation in vegetation index characteristics between tea and other landraces

**Figure 7:**
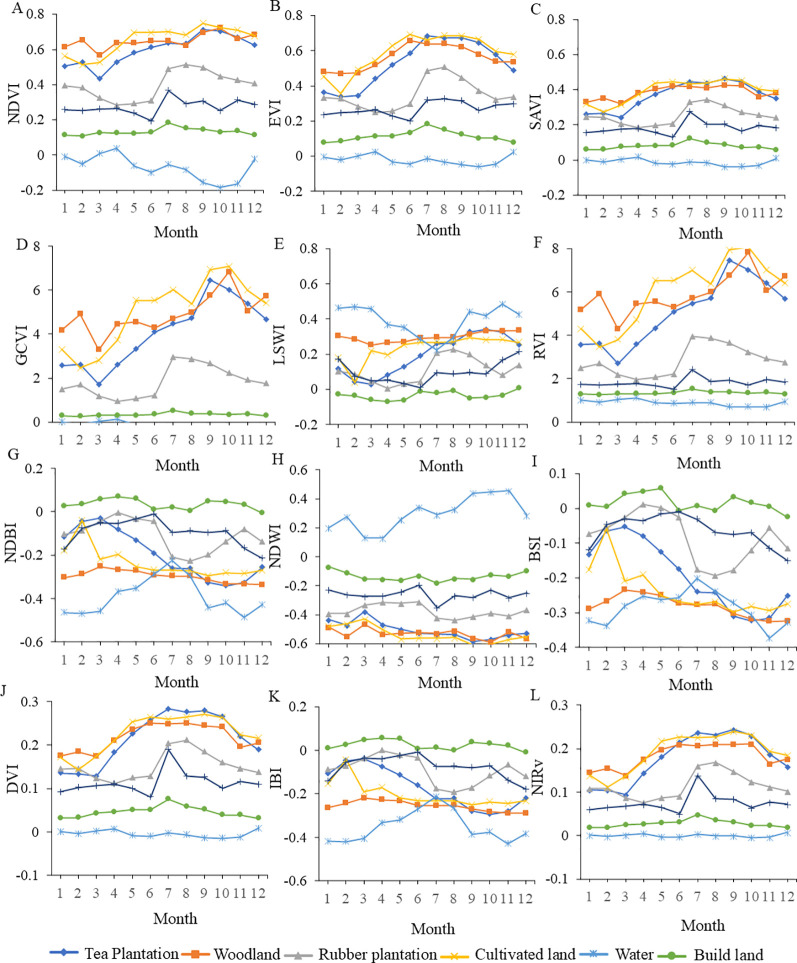
Variation of different landcover index features over phases. **(A)** NDVI; **(B)** EVI; **(C)** SAVI; **(D)** GCVI; **(E)** LSWI; **(F)** RVI; **(G)** NDBI; **(H)** NDWI; **(I)** BSI; **(J)** DVI; **(K)** IBI; **(L)** NIRv.

**Figure 6** reveals that the spectral signature curves of tea trees from October to December bear high similarity to those of rubber trees or forests, indicating that relying solely on single original spectral bands may prove insufficient for effectively distinguishing tea plantations at certain times. Spectral indices, however, encapsulate a richer array of informative content and serve as effective tools for vegetation monitoring. From a single time-phase perspective, in April, **Figure 7D** shows that tea trees can be distinguished from other land cover types in all indices except NDWI, indicating that during the spring growth peak, their biophysical characteristics significantly differ from those of non-vegetation and other vegetation types. These differences are reflected in most vegetation indices. However, it is noteworthy that confusion may arise between other land cover types, affecting overall classification accuracy.

Analyzing the ability of vegetation indices to distinguish tea trees from other land cover types across different time periods, **Figure 6I** shows that BSI exhibits strong discrimination capability for different land cover types in January, March to July, and December, highlighting the importance of BSI’s utilization of thermal infrared band information to reflect surface thermal radiation properties in crop classification. However, it is noteworthy that BSI may cause confusion between vegetation and water bodies in specific months like August and October. According to **Figure 6D** and **Figure 6F**, GCVI and RVI demonstrate good identification performance for all land cover types in January, March to May, September, October, and December, but their curves closely resemble each other, suggesting that while they have numerical differences, their differences may be reduced when analyzing land cover samples by taking the average of their vegetation index values. EVI shows distinct differences among all land cover types in March, April, May, June, and December (**Figure 6B**), while NDVI exhibits high discriminative power in January, March, April, and May (**Figure 6A**). The relevant indices presented in **Figures 6C, E, G, K, H, L**, show indistinct differentiation effects, and thus no further analysis will be conducted thereon.

### 3.3 Accuracy estimation based on single time-phase accuracy

In February, March, April, and May of 2023, the overall accuracy was relatively high, reaching 81.2%, 83.3%, 83.6%, and 79.1%, respectively, with corresponding Kappa coefficients of 0.75, 0.77, 0.78, and 0.71 (see **Table 4**). This may be attributed to the fact that after the winter dormancy period, tea tree roots absorb substantial amounts of soil nutrients. Consequently, during March and April, the reflectance characteristics of tea tree canopies significantly differ from those of general forests, leading to higher classification accuracy. Additionally, deep pruning typically occurs in mid-May following the completion of spring tea harvest. Research performed by scholars such as Yawen Kang et al. on tea tree identification in Hangzhou further corroborates the positive auxiliary role of the pruning period, a specific phenological stage, in recognizing and differentiating tea trees (Sun, 2006). Conversely, in September 2023, the overall accuracy was lowest, at just 63.2%, with a Kappa coefficient of 0.54. This may be due to the lush vegetation during summer, causing tea tree spectral curves and vegetation index values to closely resemble those of forests and other vegetation, leading to misclassification of some other land cover types as tea trees.

**Table 4:** Precision verification of classification result of tee area for different phases.

Date	Overall accuracy (%)	Producer’s accuracy (%)	User’s accuracy (%)	Kappa coefficient
2023-1	75.4	79.3	73.0	0.67
2023-2	81.2	72.3	64.9	0.75
2023-3	83.3	85.7	77.6	0.77
2023-4	83.6	87.4	78.7	0.78
2023-5	79.1	83.2	74.3	0.71
2023-6	65.5	62.4	58.8	0.59
2023-7	65.6	60.3	58.5	0.57
2023-8	65.8	59.6	58.2	0.61
2023-9	63.2	61.6	60.0	0.54
2023-10	68.7	66.4	63.8	0.57
2023-11	73.1	70.3	65.5	0.64
2023-12	72.2	75.9	70.3	0.62

According to **Figure 5B**, in February, Yunnan tea trees are still in their dormant phase, and their spectral responses may closely resemble those of other plants. Consequently, vegetation indices derived from optical remote sensing might not provide sufficient information for precise differentiation and identification of tea gardens from other vegetation types. However, utilizing Sentinel-1 satellite Synthetic Aperture Radar (SAR) data, particularly the backscatter coefficients under VV and VH polarization modes, can reveal distinct geometric structures of various land cover types. In February, tea trees within tea gardens, characterized by unique height, row spacing, and leaf arrangement patterns, generate distinctive backscatter features when illuminated by radar waves, thereby enhancing the model’s ability to identify tea garden areas. The multi-source data fusion strategy provides the model with more robust structural information support; the integration of SAR data effectively compensates for the lack of distinct spectral features during non-growing seasons (e.g., February) and data gaps in cloudy and rainy periods, playing a pivotal role in distinguishing tea trees from spectrally similar rubber plantations and natural forests, thereby significantly mitigating accuracy degradation caused by seasonal spectral confusion.

Aside from April, the VV polarization backscatter coefficient feature ranks among the top three in importance for all other months (**Figures 7A–L**). This underscores the significant role of radar polarization responses in land cover classification, as they reveal unique scattering mechanisms of crops and provide information penetrating vegetation canopies, offering indispensable SAR remote sensing support for precise identification and monitoring of different crop types and growth stages. Simultaneously, in April, spectral features and associated vegetation indices alone can achieve high-precision identification. Corresponding to springtime, vegetation experiences rapid growth, with leaf area increasing and chlorophyll content rising. Spectral features (such as visible-to-near-infrared reflectance) and vegetation indices (like NDVI and EVI) more effectively capture these changes, enhancing the distinction of vegetation cover types. Moreover, spring often entails fewer clouds and precipitation, resulting in favorable acquisition conditions for optical remote sensing data, with higher quality and usability of spectral data. Thus, the prominent role of spectral features and vegetation indices in April land cover classification indirectly highlights the significance of phenological analysis in object identification.

**Figure 8:**
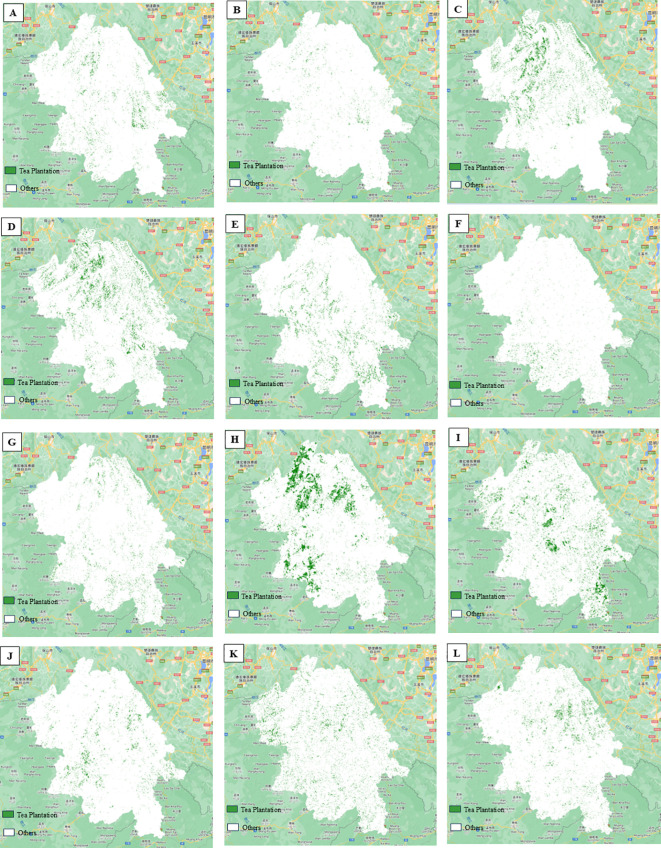
Tea distribution extraction results across different phases. **(A)** January 2023; **(B)** February 2023; **(C)** March 2023; **(D)** April 2023; **(E)** May 2023; **(F)** June 2023; **(G)** July 2023; **(H)** August 2023; **(I)** September 2023; **(J)** October 2023; **(K)** November 2023; **(L)** December 2023.

From the schematic diagrams of tea tree distribution extraction results for different time phases (**Figure 8**), considerable variations in tea tree distribution extraction outcomes across time phases can be observed. As a typical seasonal crop, tea tree growth status and biophysical properties, such as chlorophyll content, undergo significant seasonal fluctuations, directly influencing the spectral characteristics of tea trees in remote sensing imagery. During different seasons, tea tree leaf condition, coverage, color depth, and leaf surface structure vary, leading to dissimilarities in the spectral contrast between tea trees and their surroundings in remotely sensed data acquired at different times, ultimately affecting extraction accuracy and consistency.

### 3.4 Optimum features for tea plantations extraction

In this study, through spectral feature analysis, vegetation index feature analysis, and assessment of tea tree classification accuracy across different time phases, we selected March, April, and May 2023 as the best time phases for identifying tea tree crop distribution information. Following the previously established parameter configurations, we constructed a random forest classification model to extract tea tree distribution information in the southwestern part of Yunnan Province. The results showed an overall accuracy of 87.1% and a Kappa coefficient of 0.83 (see **Table 5**), indicating an improvement in classification accuracy compared to relying solely on single-time phase data. The identification of spring as the optimal phenological window for tea plantation mapping is consistent with the unique agrophysiological characteristics of *Camellia sinensis*. During early spring, tea plants break dormancy and initiate a vigorous flushing period, producing tender new leaves with distinct biophysical properties. This phenological stage is characterized by a rapid increase in chlorophyll content and leaf area index (LAI), leading to a sharp rise in near-infrared (NIR) reflectance and a pronounced drop in visible band reflectance. Crucially, this “green-up” trajectory in tea plantations often precedes or differs in magnitude from that of surrounding evergreen broadleaf forests and rubber plantations, thereby maximizing spectral separability during this narrow window. Our results confirm that leveraging this specific phenological signal significantly reduces confusion with other vegetation types compared to summer or autumn periods, where canopy structures and spectral signatures tend to converge.

**Table 5:** Precision verification of classification result of tee area.

Feature dimension	Overall accuracy(%)	Producer’s accuracy(%)	User’s accuracy(%)	Kappa coefficient
60	87.1	89.0	85.4	0.83
42	89.1	90.4	86.6	0.85

An excessive number of feature dimensions can lead to increased computational complexity and decreased classifier efficiency. Therefore, it is necessary to eliminate redundant or irrelevant features while maintaining classification accuracy without compromising feature quantity (Sun, 2006). To avoid this issue and ensure feature set simplification without compromising classification accuracy, this study, guided by the conclusions drawn from the earlier vegetation index feature analysis, purposefully removed several indices that performed poorly or contributed minimally to tea tree classification in March, April, and May, including NDWI, DVI, LSWI, NDB, IBI, and NIRv. After excluding these redundant or irrelevant features, the remaining features were used as inputs for the random forest classifier, followed by further ranking of feature importance and classification experimentation. The results demonstrated that the optimized feature set not only improved classification efficiency but also maintained classification accuracy. The final classification results revealed an overall accuracy increase to 89.1% and a Kappa coefficient rise to 0.85, with the improved classification precision indirectly substantiating the effectiveness of the random forest classification algorithm incorporating multi-time-phase and multi-feature analysis for extracting tea garden areas from medium-resolution imagery.

**Figure 9:**
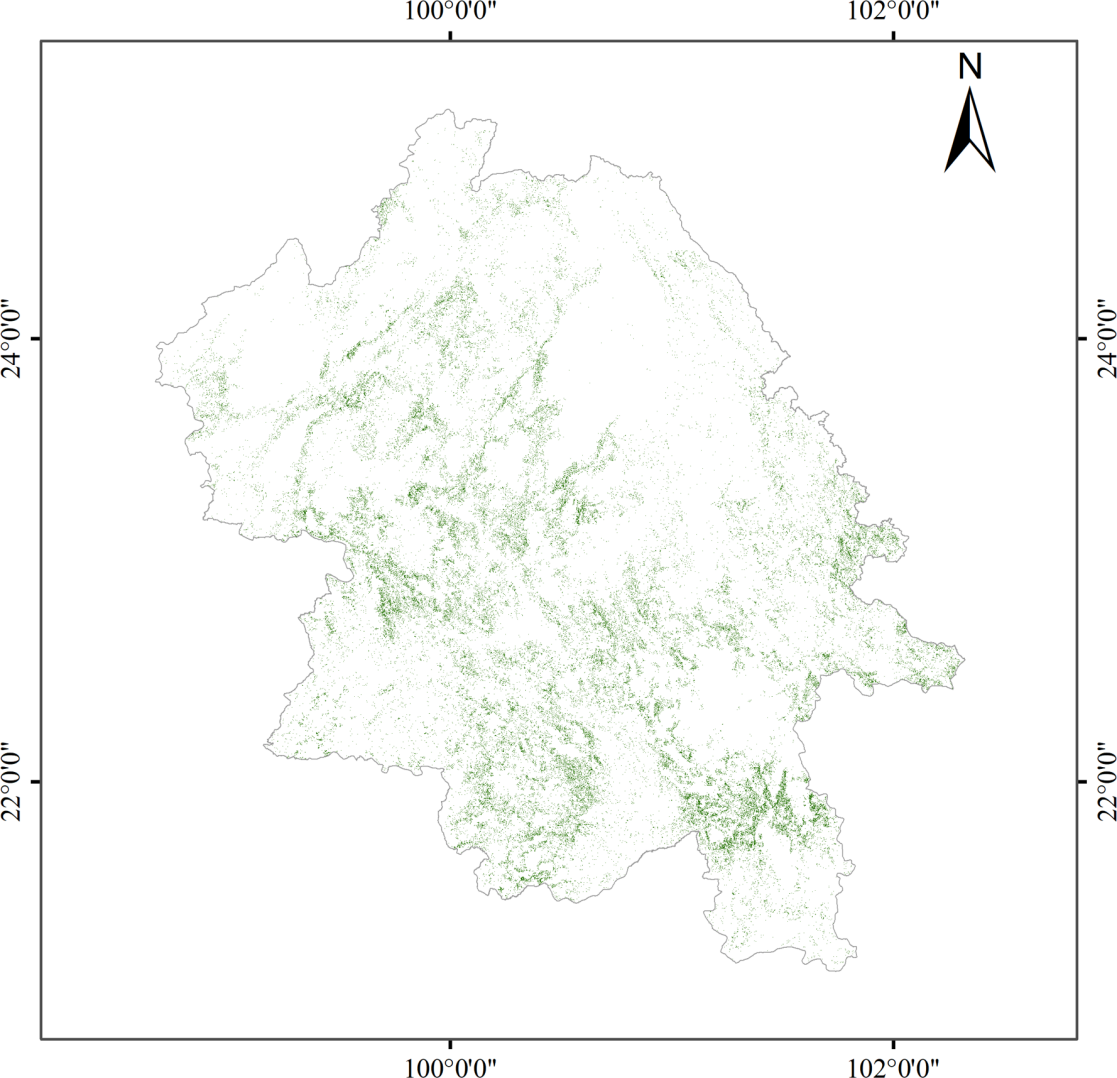
Spatial distribution of tea plantations.

Based on the results of the highest-accuracy classification following feature selection, we generated the final map depicting tea tree distribution in the southwestern part of Yunnan Province (**Figure 9**). The main distribution areas are concentrated in the low mountain and hilly terrains flanking both sides of the Lancang River. Notable tea plantations such as Fengqing Tea Garden and Menghai Tea Garden, when compared and interpreted against high-resolution satellite imagery provided by Google Earth Pro, were effectively identified. According to our study’s statistics, the total area of tea tree distribution in southwestern Yunnan amounts to approximately 3,409.73 square kilometers. However, comparing this with official statistics, the actual tea garden area within the region, as per the 2022 Statistical Yearbook of Lincang City, the 2022 Statistical Yearbook of Xishuangbanna Dai Autonomous Prefecture, and data released by the Pu’er Municipal Government, totals around 4,320.13 square kilometers. This figure exhibits a difference of approximately 910.40 square kilometers from the result obtained via remote sensing image classification in this study. While our model demonstrated high precision based on validation samples, the observed ~21% underestimation relative to statistical records likely stems from a combination of structural and methodological factors. Specifically, the prevalence of fragmented, smallholder plots scattered across Yunnan’s mountainous terrain often results in patches smaller than the sensor’s pixel resolution or isolated within forest matrices, causing them to be filtered out as noise during post-classification. Additionally, newly established or heavily intercropped tea gardens frequently lack the distinct spectral signatures of mature monocultures, closely resembling shrubs or sparse forests and thus leading to omission errors. Finally, a fundamental mismatch exists between administrative statistics, which may include abandoned, sparse, or designated-but-unplanted areas, and our remote sensing approach that strictly identifies active, spectrally distinct tea vegetation; this discrepancy is further compounded by the spectral similarity between certain shrub-like forest species and tea plants, particularly at forest margins, where conservative classification thresholds may have inadvertently excluded valid but ambiguous pixels.

However, our study acknowledges certain limitations, primarily the reliance on visually interpreted training samples from Google Earth and Baidu Maps rather than extensive *in-situ* verification, as well as the absence of empirical comparisons with alternative algorithms like SVM, XGBoost, or CNNs. To mitigate inherent uncertainties in this visual interpretation—specifically regarding seasonal dynamics and topographic shadows—we implemented a rigorous multi-temporal validation strategy during sample collection; this involved leveraging historical imagery archives to select sites with consistent tea plantation characteristics across multiple time points (with a focus on the distinct spring flushing period to distinguish tea from deciduous forests or crops) and cross-referencing ambiguous mountainous areas with Digital Elevation Models (DEMs) to differentiate shadows from actual vegetation. Although prior research (e.g., Zhu et al., 2019) indicates that Random Forest offers accuracy comparable to more complex models while providing superior computational efficiency and interpretability, the lack of direct ground-truthing remains a constraint. Consequently, future work will prioritize comprehensive field campaigns to validate and refine these remotely identified samples, alongside expanding the sampling scope to diverse regions and benchmarking against other advanced classifiers to further ensure the robustness and generalizability of our approach.

## 4 Conclusion

In the process of extracting tea tree information in Yunnan, we utilized the GEE cloud computing platform, integrating Baidu Maps API and visual interpretation for sample selection. By analyzing the spectral curve characteristics and vegetation indices of various land objects at different time phases, a random forest algorithm classifies tea trees, with the classification results verified for precision. A composite variable of multi-time-phase features, refined through feature selection, is input into the random forest model, facilitating in-depth extraction of tea tree information. This approach enables rapid testing of different feature-time-phase combinations, addressing challenges in acquiring and handling remote sensing data for tea plantation extraction. It also explores the significance of identifying optimal classification time phases for tea plantations in Yunnan Province, offering insights into overcoming challenges related to large-scale tea plantation identification with extensive time requirements. While initial progress has been made, limitations include a restricted sample set, computational resource constraints, and a singular validation approach. Future studies should focus on expanding sample sizes, diversifying sample types, enhancing computational capabilities, optimizing model architecture, and implementing multi-faceted, multi-tiered validation methods to achieve more precise and detailed investigations into crop recognition issues in both southern and northern regions.

## Data Availability

The original contributions presented in the study are included in the article/**Supplementary Material**. Further inquiries can be directed to the corresponding authors.

## Supplementary material

Supplementary Table S1 Latitude and longitude coordinates of major tea gardens in Yunnan Province sourced from Baidu Maps API.
