# Supplementary material for: Tea tree recognition based on multi-source satellite data across Southwest China
Source: Front Plant Sci. 2026 May 14;17:1801301. doi: 10.3389/fpls.2026.1801301 (PMC13216473; doi:10.3389/fpls.2026.1801301)
Supplement: Supplementary Table S1 — Latitude and longitude coordinates of major tea gardens in Yunnan Province sourced from Baidu Maps API. [file Table1.docx]

**Supplementary Materials**

**Table S1**

**Table S1.** Latitude and longitude coordinates of major tea gardens in Yunnan Province sourced from Baidu Maps API

| SN | Tea Garden Name | Longitude | Latitude | SN | Tea Garden Name | Longitude | Latitude |
| --- | --- | --- | --- | --- | --- | --- | --- |
| 1 | Puer Gaojiazhai Ten-Thousand-Mu Tea Garden | 100.98 | 22.75 | 123 | Mansong Zen Tea Yi Flavor Tribute Tea Garden Base | 101.40 | 22.15 |
| 2 | Yingpan Mountain Tea Garden Resort | 101.10 | 22.66 | 124 | Dadugang Shuangfu Tea Farm | 100.96 | 22.33 |
| 3 | Tea Garden Ecological Farmhouse | 101.13 | 22.63 | 125 | Dingjiazhai Shangzhai Bai Tea Garden | 101.49 | 22.06 |
| 4 | Menggen Tea Farm | 99.98 | 22.34 | 126 | Shanggang Village Committee Tea Farm | 101.70 | 21.31 |
| 5 | Jianli Sheng Tai Tea Garden Breeding Farm | 100.51 | 23.77 | 127 | Shangyong Village Committee Tea Farm | 101.71 | 21.25 |
| 6 | Tea Garden Villa | 101.02 | 22.77 | 128 | Manla Tea Farm Team 1 | 101.46 | 22.12 |
| 7 | Dongzhu Tea Garden Farmhouse | 99.87 | 22.66 | 129 | Longmen Village Council Tea Farm | 101.57 | 21.30 |
| 8 | Kunlu Mountain Royal Ancient Tea Garden | 101.08 | 23.25 | 130 | Xiangshan Tea Farm Team 1 | 101.32 | 22.13 |
| 9 | Manxi Tea Farm | 100.94 | 22.73 | 131 | Dadugang Tea Farm Team 12 | 100.97 | 22.36 |
| 10 | Lexin Jiuba Tea Garden | 101.10 | 22.84 | 132 | Manla Tea Farm Team 3 | 101.47 | 22.15 |
| 11 | Xidang River Shuidonggua Forest Ancient Tea Garden | 100.61 | 23.82 | 133 | Yubang Yibang Ancient Tea Garden Base | 101.36 | 22.23 |
| 12 | Small Tea Garden Restaurant | 101.09 | 23.03 | 134 | Xiangshan Tea Farm Team 2 | 101.30 | 22.13 |
| 13 | Tangwang Organic Tea Garden | 100.58 | 23.66 | 135 | Manla Tea Farm Team 2 | 101.47 | 22.11 |
| 14 | Nanjiao River Tea Farm | 99.97 | 22.52 | 136 | Longlinxuan Tea Garden Base | 101.44 | 22.00 |
| 15 | Jinggu Jiayuan Tea Farm | 100.40 | 23.76 | 137 | Xiangshan Tea Farm | 101.31 | 22.13 |
| 16 | Juwuchun Ancient Tea Garden Farmers' Professional Cooperative | 100.71 | 23.81 | 138 | Xishuangbanna Brown Tea Industry Co., Ltd. Banzhang Tea Garden | 100.47 | 21.77 |
| 17 | Yunlong Tea Farm Team 2 | 101.09 | 22.77 | 139 | Ge'er Laobanzhang Ecological Tea Garden Base | 100.47 | 21.73 |
| 18 | Kunlu Imperial Tea Garden | 101.06 | 23.05 | 140 | Mengyang Farm Tea Farm Base | 100.91 | 22.25 |
| 19 | Bangwai Xie's Ping'an Ecological Ancient Tea Garden | 99.95 | 23.13 | 141 | Yanhai Ancient Method Tea Farm | 100.81 | 22.01 |
| 20 | Jiufang Tea Farm | 99.97 | 22.47 | 142 | Ten-Thousand-Mu Tea Farm Team 2 | 101.38 | 22.10 |
| 21 | Shuifang Tea Farm | 100.05 | 22.39 | 143 | Dadugang Tea Farm Team 11 | 100.97 | 22.35 |
| 22 | Tea Garden Farmhouse | 99.94 | 22.55 | 144 | Ancient Tea Garden | 100.46 | 21.96 |
| 23 | Tea Garden Wellness Center | 101.05 | 23.07 | 145 | Tea Garden Family Tea Co., Ltd. (Menghai Branch) | 100.44 | 21.96 |
| 24 | Ninety-Three Tea Farm | 100.01 | 22.42 | 146 | Tea Garden Restaurant | 100.99 | 22.36 |
| 25 | Puer Yumei Ancient Tea Garden Professional Cooperative | 100.59 | 23.86 | 147 | Chali Ji Tea Industry Laobanzhang Tea Garden | 100.47 | 21.75 |
| 26 | Mangyun Tea Farm | 100.02 | 22.27 | 148 | Pada Ecological Tea Garden Chicken | 100.14 | 21.97 |
| 27 | Yakou Tea Farm | 100.12 | 22.28 | 149 | Baihua Mountain Tea Farm | 100.91 | 22.16 |
| 28 | Fenghuang Mountain Fenghuangwo Tea Garden | 101.38 | 23.50 | 150 | Jihaotun Shared Tea Garden - Manhei Base | 100.42 | 22.06 |
| 29 | Puer Kunlu Mountain Royal Ancient Tea Garden Co., Ltd. | 101.08 | 23.04 | 151 | Tea Garden Inside Bistro | 100.45 | 21.96 |
| 30 | Lancang Bamei Tea Farm | 99.99 | 22.36 | 152 | Dayi Tea Experience Hall · Ningjun (Mengla Road Branch) | 100.81 | 22.01 |
| 31 | Walu Tea Farm | 101.41 | 23.16 | 153 | Nannuo Mountain Banpo No.13 Tea Farmer | 100.60 | 21.96 |
| 32 | Pajing Tea Farm | 99.48 | 22.29 | 154 | Keyixing Pasha Zhongzhai Primary Processing Workshop | 100.56 | 21.83 |
| 33 | Dalongtang Tea Farm | 100.18 | 22.31 | 155 | Menghai County Pasha Chanson Manor Tea Industry | 100.56 | 21.85 |
| 34 | Gongguan Tea Farm | 101.46 | 23.37 | 156 | Songding Puer Manor | 100.47 | 21.99 |
| 35 | Fujiao Tea Farm | 99.76 | 23.02 | 157 | Luoxi Puer Tea Manor | 101.07 | 22.50 |
| 36 | Wendong Community Tea Farm | 99.92 | 23.18 | 158 | Mingsheng Puer Tea Manor | 100.44 | 21.93 |
| 37 | Laozhu Tea Farm | 101.87 | 23.06 | 159 | Yibang (Mangong) Daheishulin Cao's Tea Manor | 101.35 | 22.23 |
| 38 | Qianjiazhai Kangxing Farmhouse Ancient Tea Garden | 101.24 | 24.23 | 160 | Mingsheng Manor Puer Tea | 100.83 | 22.02 |
| 39 | Yongma Tea Farm | 101.36 | 23.36 | 161 | Dayang Tea Processing Factory (Ecological Tea Garden) | 100.93 | 22.33 |
| 40 | Nuobo Tea Farm | 99.67 | 23.20 | 162 | Xishuangbanna Menglong Duanfuxiang Tea Planting Professional Cooperative | 100.60 | 21.48 |
| 41 | Puer Tea Standard Manor | 100.09 | 22.26 | 163 | Yiwu Bohetang Wangong Ecological Tea Planting Base | 101.47 | 22.09 |
| 42 | Lan Family Old Tea Garden | 100.85 | 24.45 | 164 | Mengla Guojiashan Tea Planting Professional Cooperative | 101.62 | 22.32 |
| 43 | Rongping Tea Farm | 101.38 | 23.36 | 165 | Zhenyihao Tea Planting Professional Cooperative | 101.61 | 22.04 |
| 44 | Lancang County Xincheng Fukang Tea Farm Direct Sales Department | 100.97 | 22.75 | 166 | Mengla Yaozhu Tea Planting Professional Cooperative | 101.61 | 22.04 |
| 45 | Bailian Puer Tea Manor Experience Hall | 100.09 | 22.26 | 167 | Mengla County Xinshan Yaoxing Tea Planting Professional Cooperative | 101.56 | 21.85 |
| 46 | Tea Farm | 101.58 | 23.08 | 168 | Mengla County Zhenxin Tea Planting Professional Cooperative | 101.33 | 22.21 |
| 47 | Tea Farm | 99.39 | 22.20 | 169 | Jinghong Tea Girl Tea Planting Professional Cooperative | 100.52 | 21.50 |
| 48 | Menggen Tea Farm Team 2 | 99.99 | 22.35 | 170 | Mengla Zuixiangming Tea Planting Professional Cooperative | 101.52 | 21.91 |
| 49 | Ximeng Washan Yunwu Tea Planting Farmers' Professional Cooperative | 99.67 | 22.69 | 171 | Yibang Tianyu Tea Planting Professional Cooperative | 101.35 | 22.23 |
| 50 | Pan Hong Wanding Tea Planting Farm | 100.61 | 23.79 | 172 | Mengla Hongming Tea Planting Professional Cooperative | 101.54 | 21.82 |
| 51 | Jinggu Zongneng Tea Planting Farm | 101.09 | 23.31 | 173 | Mengla Pinzhen Tea Planting Professional Cooperative | 101.47 | 22.16 |
| 52 | Jinggu Defuyuan Tea Planting Farm | 100.69 | 23.71 | 174 | Yibang Caiyuanhao Tea Planting Base | 101.33 | 22.21 |
| 53 | Kuchongtian Tea Planting Professional Cooperative | 100.90 | 22.80 | 175 | Menghaidi Tea Planting Base | 100.47 | 21.54 |
| 54 | Fu Meizhong Tea Planting Farm | 100.63 | 23.73 | 176 | Yaoshengtang Tea Planting Professional Cooperative | 101.62 | 22.02 |
| 55 | Jinggu Kangruilin Tea Planting Base | 100.69 | 23.74 | 177 | Mansong Prince Mountain Ouyang Tea Plantation Primary Processing Workshop | 101.38 | 22.16 |
| 56 | Simao District Yingzhuo Tea Planting Farm | 100.90 | 22.86 | 178 | Beiyin Mountain Wenrong Planting and Sales Tea House | 101.44 | 22.13 |
| 57 | Jinggu Jinglong Tea Farmers' Professional Cooperative | 100.70 | 23.51 | 179 | Mingyuan Company Jinghong City Dadugang Longshan Tea Farm | 100.98 | 22.36 |
| 58 | Yunqing Tea Planting Farm | 100.44 | 23.09 | 180 | Dadugang Tea Farm Team 6 | 100.88 | 22.19 |
| 59 | Simao District Xiaoma Tea Planting Farm | 101.07 | 22.73 | 181 | Dadugang Tea Farm Team 10 | 100.97 | 22.36 |
| 60 | Jingdong Yongju Tea Industry Walnut Planting Cooperative | 100.63 | 24.44 | 182 | Molie Daliangzi Yangrui Ancient Tea Garden | 99.88 | 23.72 |
| 61 | Mojiang Ziyi Tea Planting Farm | 101.36 | 23.48 | 183 | Tea Garden Xiang Tea Workshop | 100.36 | 23.96 |
| 62 | Herun Tea Industry Natural Farming Organic Planting Base | 101.84 | 22.70 | 184 | Jinchao Ancient Tea Garden | 99.85 | 23.66 |
| 63 | Lancang Lizhahai Tea Planting Base | 99.83 | 22.88 | 185 | Nanmei Tea Garden | 99.94 | 23.96 |
| 64 | Jinggu Xuanxuan Tea Planting Farm | 100.72 | 23.74 | 186 | Lincang Senran Tea Garden | 100.33 | 23.92 |
| 65 | Luoguoshan Tea Farmers Planting Professional Cooperative | 101.82 | 22.65 | 187 | Ancient Tea Garden | 100.12 | 23.92 |
| 66 | Jinggu Xiaobao Tea Planting Farm | 100.68 | 23.74 | 188 | Erqiandao Ancient Tea Garden | 99.98 | 23.77 |
| 67 | Leiyao Tea Planting Farmers' Professional Cooperative | 101.07 | 22.76 | 189 | Bingdao Ancient Tea Garden | 99.91 | 23.79 |
| 68 | Jinggu Jiashun Tea Planting Professional Cooperative | 100.92 | 23.64 | 190 | Renshanzhishui Tea Manor | 99.96 | 24.57 |
| 69 | Xingxi Tea Planting | 101.30 | 22.66 | 191 | Shuangjiang Autonomous County Mengku Town Yunzhong Tea Manor | 99.91 | 23.78 |
| 70 | Puer Zhenyuan Miejiang Fuyou Tea Planting Professional Cooperative | 101.47 | 23.93 | 192 | Bengkong Tea Farm | 98.77 | 23.94 |
| 71 | Jinggu Yewang Tea Planting Professional Cooperative | 100.29 | 23.47 | 193 | Tea Garden Inside | 100.36 | 23.94 |
| 72 | Jinggu Daqing Qishuang Tea Planting | 100.68 | 23.74 | 194 | Chahe Tea Farm | 100.12 | 24.00 |
| 73 | Jinggu Shengjin Tea Planting Factory | 100.82 | 23.46 | 195 | Hui Ancient Tea Garden | 100.35 | 23.98 |
| 74 | Jinggu County Chen's Tea Planting Factory | 100.96 | 23.35 | 196 | Fuyichen Bingdao Laozhai Tea Garden Base | 99.91 | 23.79 |
| 75 | Jinggu Chunhang Tea Planting Professional Cooperative | 100.65 | 23.67 | 197 | Daxue Mountain Ancient Tea Garden | 100.35 | 23.97 |
| 76 | Jingdong Xingmin Tea Planting Farmers' Professional Cooperative | 100.94 | 24.31 | 198 | Bingdao Laozhai Yunzhong Tea Garden | 99.91 | 23.78 |
| 77 | Jingdong Chengyun Tea Farmers Planting Professional Cooperative | 100.72 | 24.80 | 199 | Pingcun Tea Farm | 100.28 | 23.65 |
| 78 | Zhenyuan Qingyin Tea Planting Farmers' Professional Cooperative | 101.40 | 23.99 | 200 | Bawai Laozhai Zhicheng Ancient Tea Garden | 99.93 | 23.77 |
| 79 | Nongzha River Tea Farmers Planting Professional Cooperative | 101.86 | 22.61 | 201 | Tea House Cottage | 99.26 | 24.02 |
| 80 | Qianjiazhai Zhigao Ecological Tea Planting Professional Cooperative | 101.33 | 24.09 | 202 | Tianyi Tea Source Manor | 100.09 | 23.88 |
| 81 | Lianhe Village Tea Planting Association | 101.91 | 22.73 | 203 | Bingdao Laozhai Daqiu Tea Garden | 99.91 | 23.79 |
| 82 | Jinggu Kunjianyong Tea Planting Base | 100.69 | 23.75 | 204 | Jinqiao Bingdao Ancient Tea Garden | 99.91 | 23.79 |
| 83 | Zhizun Ancient Tea Garden | 100.61 | 21.96 | 205 | Seli Tree Tea Farm | 99.07 | 24.01 |
| 84 | Bulang Mountain Tea Base | 100.46 | 21.75 | 206 | Mengjiao Tea Farm | 99.24 | 23.24 |
| 85 | Baicaotang Ecological Tea Garden Company | 100.73 | 22.07 | 207 | Nuoliang Tea Farm | 99.34 | 23.22 |
| 86 | Mingxiu Ancient Tea Garden | 100.57 | 21.49 | 208 | Tuanshan Tea Farm | 99.04 | 23.88 |
| 87 | Nannuo Mountain Banpo Laozhai Ancient Tea Garden | 100.61 | 21.95 | 209 | Tea Forest Tea Farm | 98.92 | 24.00 |
| 88 | Yunli Yunwai Tea Garden | 100.96 | 22.34 | 210 | Yongnong Tea Farm | 99.55 | 23.17 |
| 89 | Dadugang Tea Farm | 100.94 | 22.37 | 211 | Lianhe Tea Farm | 99.62 | 23.21 |
| 90 | Zhenshang Tea Garden | 100.45 | 21.97 | 212 | Mengdi Farm Tea Farm | 99.45 | 24.19 |
| 91 | Yilanya Ecological Tea Garden | 100.48 | 21.99 | 213 | Yong'an Tea Farm | 99.32 | 23.38 |
| 92 | Dadugang Ten-Thousand-Mu Tea Garden | 100.97 | 22.36 | 214 | Banlie Tea Farm | 99.25 | 23.36 |
| 93 | Yunnan Menghai Muyihao Ancient Tea Garden Co., Ltd. | 100.46 | 22.01 | 215 | Yongshuai Tea Farm | 99.61 | 23.20 |
| 94 | Dadugang Tea Farm Team 14 | 100.96 | 22.34 | 216 | Yongdong Tea Farm | 99.42 | 23.20 |
| 95 | Sanjun Tea Industry Manor | 100.62 | 21.93 | 217 | Sanjiao Mountain Tea Farm | 99.13 | 23.83 |
| 96 | Menghai Bafang Tea Garden Tea Industry Co., Ltd. | 100.50 | 22.00 | 218 | Yunxian Manwan Town Baiying Mountain Wuji Tea Manor | 100.34 | 24.63 |
| 97 | Nuogan Ancient Tea Garden | 100.83 | 21.99 | 219 | Yingshan Tea Farm | 99.66 | 23.28 |
| 98 | Zhongyixiang Brand Ancient Tea Garden Base | 100.61 | 21.95 | 220 | Fugong Tea Farm | 99.13 | 23.32 |
| 99 | Tea Garden | 100.94 | 22.37 | 221 | Wennai Dacha Valley Tea Industry Manor | 100.21 | 24.27 |
| 100 | Tea Garden | 100.39 | 21.85 | 222 | Yunxian Xianming Tea Planting Professional Cooperative | 100.34 | 24.64 |
| 101 | Bankainan Ancient Tea Garden | 100.51 | 21.79 | 223 | Lincang City Linxiang District Maohong Tea Planting Professional Cooperative | 100.30 | 23.86 |
| 102 | Fanpu Guizhen Tea Garden | 100.61 | 21.88 | 224 | Lincang Shangpin Tea Planting Farmers' Professional Cooperative | 100.30 | 24.05 |
| 103 | Xinjing Tea Garden | 100.45 | 21.97 | 225 | Linxiang District Bangdong Township Yonghe Tea Planting Farm | 100.37 | 23.90 |
| 104 | Rainforest Ancient Tea Workshop | 100.57 | 22.05 | 226 | Lincang Mingwu Tea Planting Professional Cooperative | 100.40 | 24.53 |
| 105 | Dayi Tea Manor Tea Experience Center | 100.43 | 22.00 | 227 | Fengqing County Guanzhi Tea Planting Professional Cooperative | 99.82 | 24.57 |
| 106 | Banpen Laozhai No.77 Ancient Tea Tree Garden | 100.53 | 21.74 | 228 | Fengqing County Shuangde Tea Planting Professional Cooperative | 99.78 | 24.51 |
| 107 | Puwen Dongding Tea Farm | 101.07 | 22.52 | 229 | Linxiang District Yanbang Tea Planting Farmers' Professional Cooperative | 100.35 | 23.94 |
| 108 | Laoxiang River Ecological Tea Garden | 101.63 | 21.94 | 230 | Lincang City Linxiang District Hongyan Tea Planting Farmers' Cooperative | 100.11 | 23.62 |
| 109 | Dadugang Tea Farm Team 1 | 100.91 | 22.27 | 231 | Zhenkang Qifeng Tea Planting Professional Cooperative | 98.97 | 23.99 |
| 110 | Zengming Chahao Nannuo Mountain Tea Garden Base | 100.61 | 21.97 | 232 | Yongde County Fuguang Tea Planting Professional Cooperative | 99.29 | 24.20 |
| 111 | Xiawu Tea Garden | 100.48 | 22.01 | 233 | Yongde County Mingrenyun Tea Planting Professional Cooperative | 99.64 | 24.23 |
| 112 | Bafang Tea Garden (Xingfu Road Branch) | 100.45 | 21.97 | 234 | Lincang Changnianfang Tea Planting Professional Cooperative | 100.32 | 24.05 |
| 113 | High Altitude Shatu Ancient Tea Garden | 100.82 | 22.01 | 235 | Lincang City Linxiang District Xiaoshantou Tea Planting Farmers' Professional Cooperative | 100.17 | 23.72 |
| 114 | Daqiaotou Tea Garden | 101.32 | 22.22 | 236 | Dahusai Jinbiao Tea Planting Professional Cooperative | 99.89 | 23.64 |
| 115 | Yibang Mangong Old Street Peng's Ancient Tea Garden | 101.36 | 22.23 | 237 | Cai's Tea Family Tea Planting Professional Cooperative | 99.94 | 24.72 |
| 116 | Ten-Thousand-Mu Tea Garden Puer Tea Manor | 101.00 | 22.36 | 238 | Lincang City Linxiang District Chengjun Tea Planting Professional Cooperative | 100.11 | 23.87 |
| 117 | Yizhongyun Ancient Tea Tree Manor | 100.56 | 22.07 | 239 | Lincang City Linxiang District Bangdong Manglai Hongcheng Tea Planting Professional Cooperative | 100.36 | 23.94 |
| 118 | Yuangong Bridge Tea Garden Tea House | 101.47 | 22.11 | 240 | Fengqing County Jianshan Tea Planting Professional Cooperative | 99.90 | 24.64 |
| 119 | Dingjiazhai Wangong River White Tea Garden | 101.49 | 22.06 | 241 | Lincang City Linxiang District Bangman Guqing Farmers' Professional Cooperative | 100.36 | 23.96 |
| 120 | Yiwu Mapazhai Yaoshan Tea Garden Ecological Pure Material | 101.58 | 22.28 | 242 | Fengqing Kanghong Chinese Herbal Medicine Planting | 99.98 | 24.56 |
| 121 | Mansong Prince Mountain Mangong Imperial Tea Garden Base | 101.37 | 22.14 | 243 | Tea Farm | 99.52 | 23.26 |
| 122 | Yibang Imperial Tea Garden Primary Processing Workshop | 101.32 | 22.21 |  |  |  |  |
